# Epidemiology and Sequence-Based Evolutionary Analysis of Circulating Non-Polio Enteroviruses

**DOI:** 10.3390/microorganisms8121856

**Published:** 2020-11-25

**Authors:** David M. Brown, Yun Zhang, Richard H. Scheuermann

**Affiliations:** 1 Department of Synthetic Biology, J. Craig Venter Institute, Rockville, MD 20850, USA; dbrown@jcvi.org; 2 Department of Informatics, J. Craig Venter Institute, La Jolla, CA 92037, USA; yun.zhang@jcvi.org; 3 Department of Pathology, University of California San Diego, La Jolla, CA 92093, USA; 4 La Jolla Institute for Immunology, La Jolla, CA 92065, USA

**Keywords:** enterovirus A71 (EV-A71), coxsackievirus A16 (CV-A16), coxsackievirus A6 (CV-A6), Echovirus 30 (E30), coxsackievirus A24 (CV-A24), enterovirus D68 (EV-D68), hand, foot, and mouth disease (HFMD), acute flaccid myelitis (AFM), acute flaccid paralysis (AFP), Virus Pathogen Resource (ViPR)

## Abstract

Enteroviruses (EVs) are positive-sense RNA viruses, with over 50,000 nucleotide sequences publicly available. While most human infections are typically associated with mild respiratory symptoms, several different EV types have also been associated with severe human disease, especially acute flaccid paralysis (AFP), particularly with endemic members of the EV-B species and two pandemic types—EV-A71 and EV-D68—that appear to be responsible for recent widespread outbreaks. Here we review the recent literature on the prevalence, characteristics, and circulation dynamics of different enterovirus types and combine this with an analysis of the sequence coverage of different EV types in public databases (e.g., the Virus Pathogen Resource). This evaluation reveals temporal and geographic differences in EV circulation and sequence distribution, highlighting recent EV outbreaks and revealing gaps in sequence coverage. Phylogenetic analysis of the EV genus shows the relatedness of different EV types. Recombination analysis of the EV-A species provides evidence for recombination as a mechanism of genomic diversification. The absence of broadly protective vaccines and effective antivirals makes human enteroviruses important pathogens of public health concern.

## 1. Introduction

Enteroviruses (EVs) are members of the *Picornaviridae* family of small (30 nm diameter virions) non-enveloped viruses. The enterovirus genome is a single-stranded positive-sense RNA molecule of approximately 7500 nucleotides, containing a 5′ untranslated region (UTR) of approximately 750 nucleotides and a short 3′UTR of approximately 70–100 nucleotides [1]. Enteroviruses include important human pathogens, including polioviruses, rhinoviruses, echoviruses, and coxsackieviruses. Established in 1988, the World Health Organization (WHO) Global Polio Eradication Initiative (GPEI) contributed to an initial wealth of information on various enteroviruses [2]. In addition to polio surveillance, non-polio enteroviruses are passively surveilled across several governmental and intergovernmental agencies, primarily across the Asia–Pacific region, Europe, and the United States. Enteroviruses typically cause mild respiratory and/or gastrointestinal diseases. However, they can also be associated with severe disease, even in otherwise healthy individuals [1]. Here we review the epidemiology of different enterovirus types reported in the literature, compare these epidemiological patterns with the extent of genome sequence coverage in public databases, and assess the evolutionary relatedness of different enterovirus types using genomic analysis. We have focused on non-polio and non-rhino enteroviruses, especially those associated with neurological disorders. The effectiveness of the GPEI and challenges with achieving final complete eradication of poliovirus, and the endemic nature and broad geographic distribution of rhinoviruses have been reviewed elsewhere [3,4,5,6,7].

## 2. EV Classification and Nomenclature

Enteroviruses are classified into 15 species by the International Committee on Taxonomy of Viruses (https://talk.ictvonline.org)—enterovirus (EV) A through L and rhinovirus (RV) A through C. RV-A, B, and C and EV-A, B, C, and D are the only enterovirus species that infect humans. Enterovirus subspecies were originally classified serologically based on cross-neutralization testing and were referred to as “serotypes”. More recently, due to the widespread use of genome sequencing for virus characterization, subspecies classification is now based on the sequence of the major hypervariable capsid protein VP1 and they are referred to as “types”. To date, 326 enterovirus types have been identified across the 15 different species. Among the different species that infect humans, these include 168 different rhinovirus types, 3 poliovirus types, and 103 other enterovirus types [8]. Enterovirus types are defined based on > 75% nucleotide and >85% amino acid sequence identity to a prototype strain [9], although some isolates within a type have drifted slightly beyond these thresholds [10]. New enterovirus types are designated using the prefix EV or RV, followed by the species letter and a type number designation. For historical reasons, some enterovirus types have been referred to differently. Echoviruses are all part of the enterovirus B species; however, they are abbreviated with the prefix “E” (e.g., E30) even though they are not members of the enterovirus E species. Specific coxsackievirus (CV) types are designated with the CV prefix and can belong to either EV-A, EV-B, or EV-C species depending on their VP1 sequence characteristics.

## 3. Enterovirus Association with Severe Disease

Most EVs enter the body via the digestive or respiratory tracts and are transmitted by fecal–oral or respiratory routes [1]. Fortunately, most EV infections appear to be asymptomatic [11]. For example, it has been estimated that up to 72% of poliovirus infections are resolved without symptoms [12]. Interestingly, over 10% of healthy children excrete enterovirus in their stool [13]. When symptomatic, enterovirus infections typically cause mild symptoms not requiring hospitalization [14]. However, some EV infections are associated with severe disease, in which symptoms may include acute flaccid paralysis (AFP), herpangina, hepatitis, pleurodynia, diarrhea, fever, upper and lower respiratory disease, myocarditis, pericarditis, conjunctivitis, meningitis, encephalitis, pancreatitis, and possibly diabetes [15]. Numerous circulating enterovirus types have been identified from patients with severe disease, with variable correlation between type and symptoms. For example, although EV-A types are more often associated with hand, foot, and mouth disease (HFMD), EV-B types have also been identified through HFMD [15,16]. In addition, nearly every enterovirus type has been identified from AFP patients, but the strength of association is much stronger with some types [17].

### 3.1. Enterovirus A

Enterovirus A types are the most common cause of HFMD epidemics. HFMD is characterized by painful sores that can develop in the mouth (herpangina), as well as skin rashes on the palms of the hands and soles of the feet. Initial symptoms include fever, sore throat, and malaise. HFMD epidemics have been increasingly reported across the Asia–Pacific region, in particular China [18,19], where approximately 1.3 million cases of HFMD were reported in 2009, and that number continued to rise until 2014, when over 2.7 million cases of HFMD were reported [20]. Over 2 million cases per year have been reported since then. HFMD outbreaks have also been reported in Japan [21], Taiwan [22], Hong Kong [14], Malaysia [23], Singapore [24], Vietnam [25,26,27], Thailand [28,29] and Cambodia [30]. Because of these widespread outbreaks, many East Asian countries now have sophisticated HFMD reporting systems [19,31,32], and therefore most sequences of enterovirus A are clinical samples from HFMD-afflicted patients.

CV-A16 infection is typically characterized with mild symptoms. Its incidence of infection has remained relatively flat. However, the emergence of a new clade [33] in China indicates that CV-A16 is continuing to evolve into more diverse branches, so continued surveillance is warranted. The prevalence of CV-A6 and CV-A10 have increased significantly in the recent past [34] with CV-A6 becoming the most common causes of HFMD worldwide [35,36,37]. This rise in CV-A6 prevalence coincided with an increased proportion of the D3 clade [38], suggesting an increase in transmissibility. Unfortunately, different EV-A types exhibit different antigenic properties such that little cross-reactivity between types is observed. Recurrent HFMD is almost always caused by another enterovirus type [39].

EV-A71, one of the most well-studied enteroviruses, further grouped into three main genotypes—A (the prototype), B, and C—with some newly-identified genotypes (D, E, and F) recently described [40]. EV-A71 was the most common cause of HFMD outbreaks during the 2007–2012 period, in 31 of 71 (44%) outbreaks, in some cases causing over 80% of cases [16]. EV-A71 has been implicated in up to 96% of severe cases of HFMD in some outbreaks [41,42,43]. EV-A71 has also been implicated in several AFP outbreaks across Asia and Australia [44,45,46,47,48,49]. Cell culture and animal models of EV-A71 [50,51] exist and have been used to study an extensive set of different genetic determinants of virulence [52].

As of December 2015, an EV-A71 vaccine has been available in China [53,54]. It has also been reported that the vaccine provides 80% protection against severe HFMD and 90% protection against mild HFMD [54]. The effects of this new vaccine and others on viral evolution and epidemiology remain to be determined. However, with a decreasing proportion of HFMD outbreaks attributed to EV-A71, additional vaccination against CV-A6 in particular may be needed.

### 3.2. Enterovirus B

Enterovirus B is a broad and diverse species that includes 59 different types. Acute flaccid paralysis (AFP) is one of the most serious conditions attributed to enterovirus B. In a systematic review of the literature, enterovirus B types have been implicated in 72% of all enterovirus-associated AFP cases [17], with some types only found sporadically and others more commonly, emphasizing both the commonality and diversity of AFP association. EV-B viruses generally replicate well in cell culture and thus are more likely to be identified from a particular AFP case. E11 was the most common EV-B type found from AFP surveillance. However, it was only implicated in 6.8% of AFP cases. Recent AFP surveillance reports from China, Spain, and West Africa agree with these previous findings, with 100%, 81% and 90% of positive EV samples from AFP patients belonging to the EV-B species, respectively [55,56,57,58].

Echovirus 30 is a common enterovirus B type and is the principle cause of viral meningitis and encephalitis in temperate climates, particularly in Europe. Recent outbreaks have occurred in Europe, the Americas, and the Asia–Pacific. Numerous other EV-B types have also been associated with severe diseases, including encephalitis and meningitis [15]. Myocarditis has been associated with a number of EV-B types, most significantly the CV-Bs [59,60], along with 12 other EV-B types. The high incidence of CV-B 1-6 with myocarditis, along with their association with pancreatitis [61] may reflect their common phylogeny.

### 3.3. Enterovirus C

Non-polio enterovirus C types are relatively uncommon. CV-A24 has been responsible for widespread outbreaks of conjunctivitis—an inflammation of the outer membrane of the eyelid [62,63,64]. CV-A24 spreads mainly through contact with eye secretions. CV-A24 infections are mainly observed in the tropics during the hot, rainy season, including recent outbreaks in China, French Guiana, India, Brazil, and Western Africa [64,65,66,67,68,69]. Other EV-C types can cause other diseases, including AFP, encephalitis, meningitis, HFMD, and respiratory diseases, but are not a major cause of recent cases.

### 3.4. Enterovirus D

Enterovirus D infections were not frequently reported prior to 2012. However, that changed with the recent EV-D68 biennial outbreaks starting in 2014. EV-D68 was first detected as a respiratory virus in children with pneumonia and bronchiolitis in 1962 [70]. Since then, a number of distinct clades have evolved and are currently co-circulating worldwide [71]. In the summer and fall of 2014, North America experienced a widespread outbreak of severe respiratory illness associated with EV-D68, with smaller clusters of infections also reported in Europe and Asia, resulting in the public health community classifying EV-D68 as a re-emerging pathogen of public health concern [72]. EV-D68 cases peaked again in 2016 and 2018 following a biennial pattern typical of some enterovirus types, particularly in the US [73,74,75,76,77,78,79,80], but also in South America [81] and Europe [82,83,84]. Interestingly, EV-D68 has also followed a biennial pattern in Japan, but instead peaked in 2013 and 2015 [85].

In the 2014 outbreak, reports of acute flaccid myelitis (AFM) (AFP with radiographic evidence of spinal cord inflammation) in some children with detectable EV-D68 raised concerns that genetic changes in EV-D68 could be contributing to increased disease severity and neurological symptoms [86]. AFM cases in Colorado [87] and California [88] both suggested that the number of AFM cases was significantly higher during the 2014 EV-D68 outbreak in comparison with historical controls. Among these AFM cases, EV-D68 infection was confirmed in many independent epidemiological clusters in several US states [87,89,90,91,92,93], Europe [94,95], and Australia [96]. Evidence has been mounting that EV-D68 is the cause of these recent AFM clusters. Pan-viral serology implicated enteroviruses in AFM [97]. A report on the Bradford Hill criteria for establishing a causal relationship in human infectious diseases showed that EV-D68 fulfills seven of the nine criteria [82]. In particular, the co-clustering of EV-D68 infections and AFM in the summer/fall of the 2014, 2016, and 2018 outbreaks [78,98,99] suggests a causal relationship. As of 31 August 2020, a 2020 outbreak has not been detected in the United States, possibly due to the social distancing and infection control measures implemented in response to the COVID-19 pandemic (https://www.cdc.gov/acute-flaccid-myelitis/).

A phylogenetic and comparative genomic analysis of EV-D68 [11] identified 21 unique substitutions in the novel B1 clade isolates that distinguished them from other EV-D68 isolates. Interestingly, at 12 of these positions, B1 isolates carry the same amino acid and nucleotide residues observed at equivalent positions in other paralysis-causing enteroviruses, suggesting that unique B1 substitutions may be responsible for the apparent increased incidence of neuropathology associated with the 2014 outbreak [11]. A subset of these substitutions, in particular six of the twelve coding mutations—polyprotein M291T (VP2 222), V341A (VP3 24), T860N (VP1 308), D927N (2A 66), S1108G (2C 1) and R2005K (3D 274) [90,100]—associated with neurovirulence in US strains were not found in Chinese strains [100,101]. This might explain the lower pathogenicity of Chinese strains and/or the lower relative prevalence of EV-D68 in China. Recently established mouse and human neuronal cell models [102,103] may provide systems to test novel therapeutics, or whether particular genetic residues are responsible for neurotropism.

During the 2014 outbreak, an interclade recombination was observed between the B1 and B2 subclades [104]. Sequence analysis has led to the classification of a new clade D (formally Clade A2) [105,106]. The D1 clade has become prominent in France [83] and Italy [84] in the most recent 2018 outbreak. Recombination between clade D1 and A has also been observed [106]. The novel B3 clade emerged following the worldwide outbreak in 2016 [100,105,107] and has been associated with neurological symptoms in Sweden [108], the Netherlands [109], Italy [110], and the United States [111]. Interestingly, the six common mutations (M291T, V341A, T860N, D927N, S1108G, and R2005K) associated with neurovirulence in the 2014 US B1 outbreak strains [11,90,112] are not present in B3 isolates from 2016, suggesting that either the genetic residues responsible for neurovirulence have yet to be identified or that convergent evolution result in multiple paths to neurovirulence. Indeed, at least some strains of clade D, B1 and B2 have been shown to be neurotropic [80].

EV-D68 is primarily a respiratory virus causing nasal congestion, cough, sore throat, and fever [113,114]. Its replication cycle primarily occurs in the nasal cavity, and unlike other enteroviruses is only very rarely detected in stool samples [115], thus evading detection from most AFM surveillance studies. EV-D68 presence in CSF may also be rarely detected due to the possibility of retrograde axonal transport. Indeed, EV-D68 was not be detected in CSF from AFM patients with positive NP swabs [74]. This is not uncommon with other enteroviruses, likely because of low viral loads in the CSF [115], and so diagnostic criteria recommendations now include the collection of both respiratory and stool samples. Consistent with EV-D68 infection, the CDC has reported that 90% of US AFM cases involved patient with prior respiratory symptoms; however, only 22% of respiratory samples in 2018 AFM cases carried detectable EV-D68, revealing a likely and important gap in detection capacity for EV-D68 [78].

## 4. Enterovirus Evolutionary Relatedness

**Figure 1:**
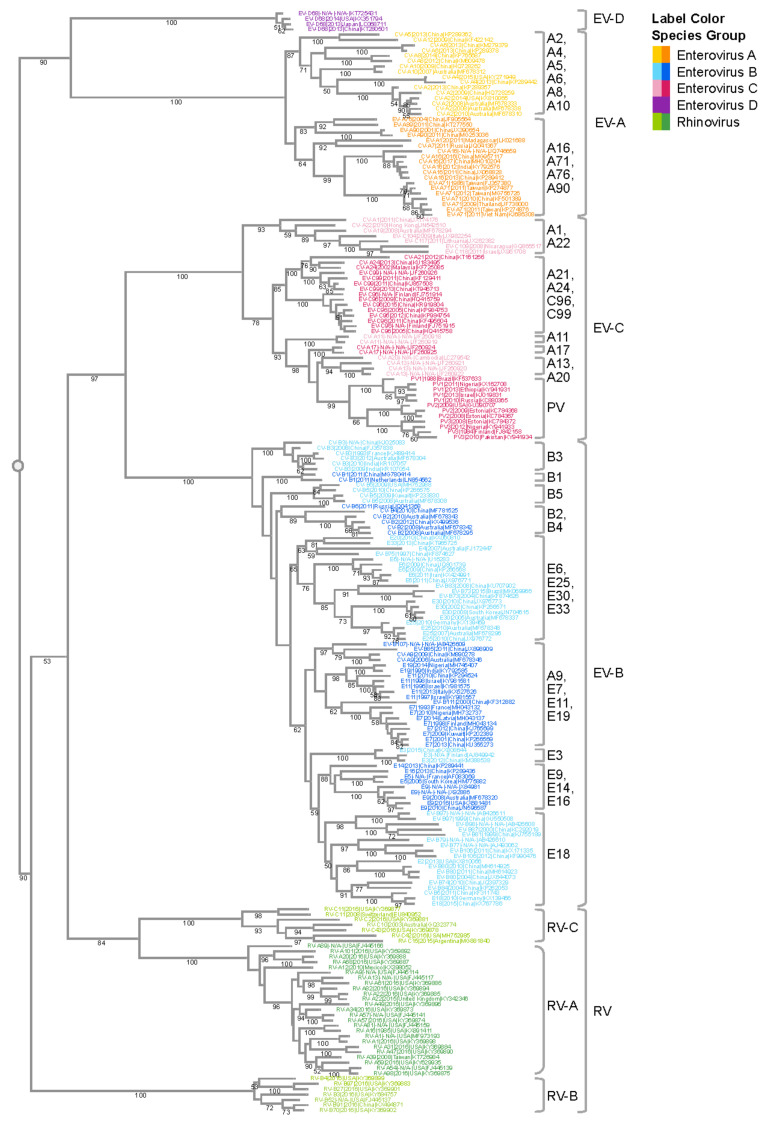
Phylogenetic analysis of the enterovirus genus. VP1 gene sequences from complete human enterovirus genomes were retrieved from the ViPR database (https://www.viprbrc.org/). Sequences representative of the genomic diversity were selected using CD-HIT [128], with a threshold of 0.90, and subsequently aligned using MAFFT E-INS-i. The phylogenetic tree was generated using RaXML with 100 bootstrap replicates, and then visualized in Archaeopteryx.js on ViPR with leaf nodes color coded by phylogenetic relatedness. Bootstrap support values of 50 or larger are shown in the tree. Major monophyletic groups are labeled with the major types in the group. Major types are defined as those with 50 or more genomes in the case of EV-A and EV-C, and 200 or more genomes in the case of EV-B.

Like other positive-sense RNA viruses, enteroviruses encode a polymerase that lacks a proof-reading mechanism [116], and they are one of the most rapidly evolving virus groups [117], with a relatively high substitution rate of 5–12 × 10^−3^ substitutions per nucleotide per year (s/n/y) [10,118]. We performed a phylogenetic analysis of VP1 nucleotide sequences which illustrates the species and subspecies/type relationships of human enterovirus isolates (Figure 1). The four human EV species (A–D) and the three RV species (A–C) are well segregated with 100% bootstrap support values. Isolates of the EV-D species are more similar to EV-A isolates than isolates of the other enterovirus species, whereas EV-C and EV-B isolates are more similar to each than to isolates of the other species, in agreement with previous reports [15]. Due to the lack of whole-genome sequences for EV-D70 and EV-D94, the EV-D species is currently only represented by a single type—EV-D68—in this phylogenetic analysis. Type relationships are also reflected in the branching structures with independent isolates of a given EV type closely connected with short branch lengths. For example, all six CV-B3 isolates are located within the same phylogenetic branch even though they come from diverse geographic regions (India, China, Australia, and France). The tree also highlights the challenge with the historical nomenclature in that echovirus and coxsackievirus types are interspersed in the phylogenetic tree. For example, CV-A9 is quite similar in sequence to E19, while quite different from CV-A10. Thus, the current nomenclature that distinguishes enteroviruses, echoviruses, and coxsackieviruses does not reliably reflect the evolutionary relatedness illustrated in the phylogenetic analysis of VP1 and other genomic regions.

Within the EV-A subtree, CV-A16 and EV-A71 are closely related to each other, forming a monophyletic group with a support value of 99%. The genetic similarity between these two types is likely responsible for the fact that both cause HFMD. On the other hand, the clear segregation of CV-A16 and EV-A71 in the tree also correlates with their distinct clinical characteristics. CV-A16 infections are usually mild, while cases of HFMD associated with severe neurological complications are usually associated with EV-A71 infections. Surveillance studies on HFMD outbreaks found that CV-A16 and EV-A71 often co-circulate [24,119], which could be the result of a common evolutionary origin. Co-circulation of these viruses further provides an ecological niche for intertypic recombination, resulting in intertwined evolutionary trajectories and emergence of new enterovirus variants. For example, a novel recombinant EV-A71 virus was found to be responsible for a large HFMD outbreak in China in 2008 [120].

Another monophyletic group within the EV-A subtree includes CV-A2, CV-A4, CV-A5, CV-A6, and CV-A10. Compared with other groups of similar sizes, this group has a larger number of types. This is likely the outcome of finer granularity of virus classification, rather than more genetic diversification. All virus types within this subtree are notably distinct from each other, as shown by the type-level clustering with 100% support values. Among the various types, CV-A2, CV-A6, and CV-A10 have been increasingly associated with HFMD, herpangina, and onychomadesis [34,35,36,37,121]. Similar to the CV-A16/EV-A71 group, CV-A6 and CV-A10 often co-circulate. Furthermore, it has been reported that CV-A6, CV-A10, CV-A16, and EV-A71 co-circulate together [122], which helps to shape the interacting evolution of these viruses.

EV-B has abundant virus types, and each type forms its own distinct cluster in the EV-B subtree. Compared with EV-A and EV-C, different EV-B types are more similar to each other. Consequently, not all intertypic clusters in this subtree have strong support values. One of the major pathogens in EV-B is E30, which has caused large outbreaks in Asia, Europe, and Americas. The four E30 strains in the subtree form a tight cluster (bootstrap = 100%) with diverse year coverage. Studies have shown the independent evolution of structural and non-structural gene regions in EV-B [123]. The VP1 region experiences progressive drift as lineages emerge, dominate, and then are replaced by new lineages over periods of 3–5 years. The temporal diversity in the E30 cluster is congruent with the lineage replacement model. It should be noted that the percentage of complete genomes/all genomes of EV-B is significantly lower (1.8%) than that of EV-A (5.2%), EV-C (5.5%) or EV-D (12.1%). Due to the small number of complete genomes available for E30, the E30 sequences used in this study may not adequately reflect the overall type diversity, even though they still cover the Asia–Pacific and America regions, and different years. With this limitation in mind, the representative E30 sequences selected by clustering still show a temporal rather than geographic relationship, which can be explained by the time-related turnover model [123].

The EV-C subtree has three major monophyletic groups. The poliovirus group is farther away from the rest of the EV-C viruses, which is consistent with the fact that poliovirus has higher incidence of paralysis than other types. CV-A21, CV-24, EV-C96, and EV-C99 belong to the same group. Even though they are genetically close, they exhibit a wide spectrum of clinical manifestations, with CV-A24 causing conjunctivitis [62,63,64], and C96/C99 associated with AFP and gastroenteritis [124].

Natural recombination among enteroviruses is frequent, occurring every few years within a species [123,125,126,127]. Recombination appears to occur either by a template switching mechanism [128,129] in which the RNA-dependent RNA polymerase switches from one RNA genome template to another in a co-infected cell, or by a non-replicative breaking–joining mechanism [130,131], in which viral RNAs are cleaved and their exposed ends rejoined. Numerous intra-species recombination events have occurred among enteroviruses A–D [132]; occasionally, interspecies recombination has occurred, usually within the 5′UTR [133,134,135]. Both recombination and spontaneous mutations can impact virulence and tissue tropism in EV strains, particularly due to effects of sequence variations in the 5′-UTR, VP1, 2A, 3C, and 3D proteins [51,52].

**Figure 2:**
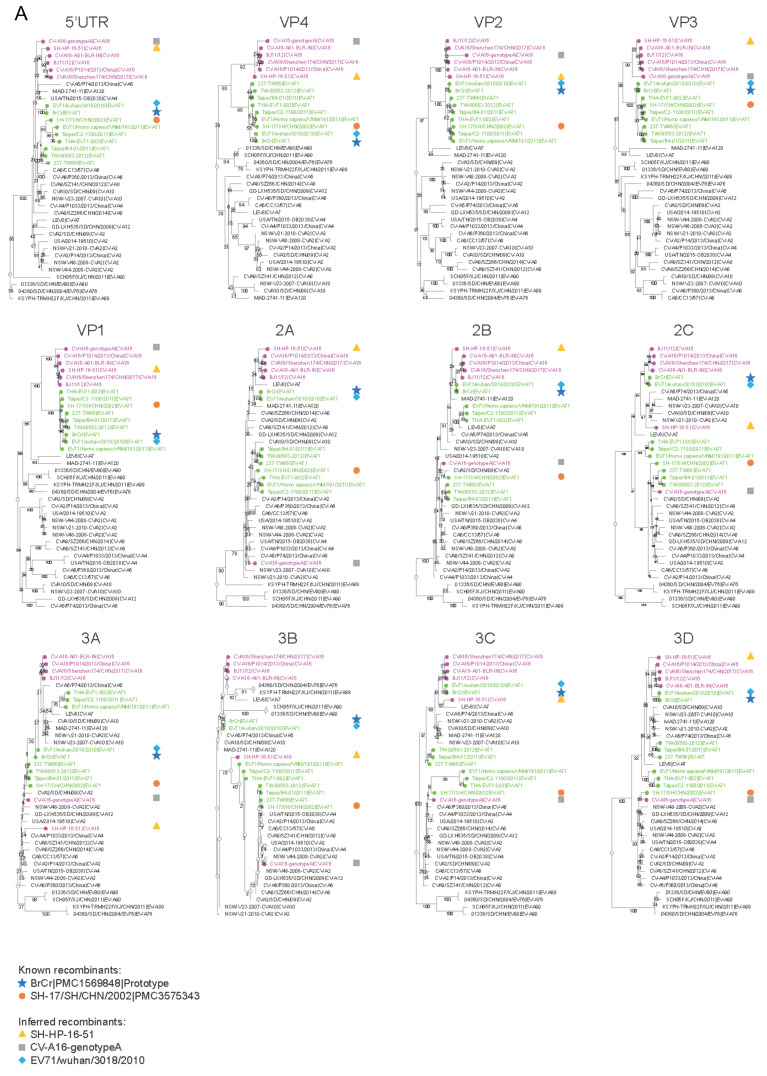

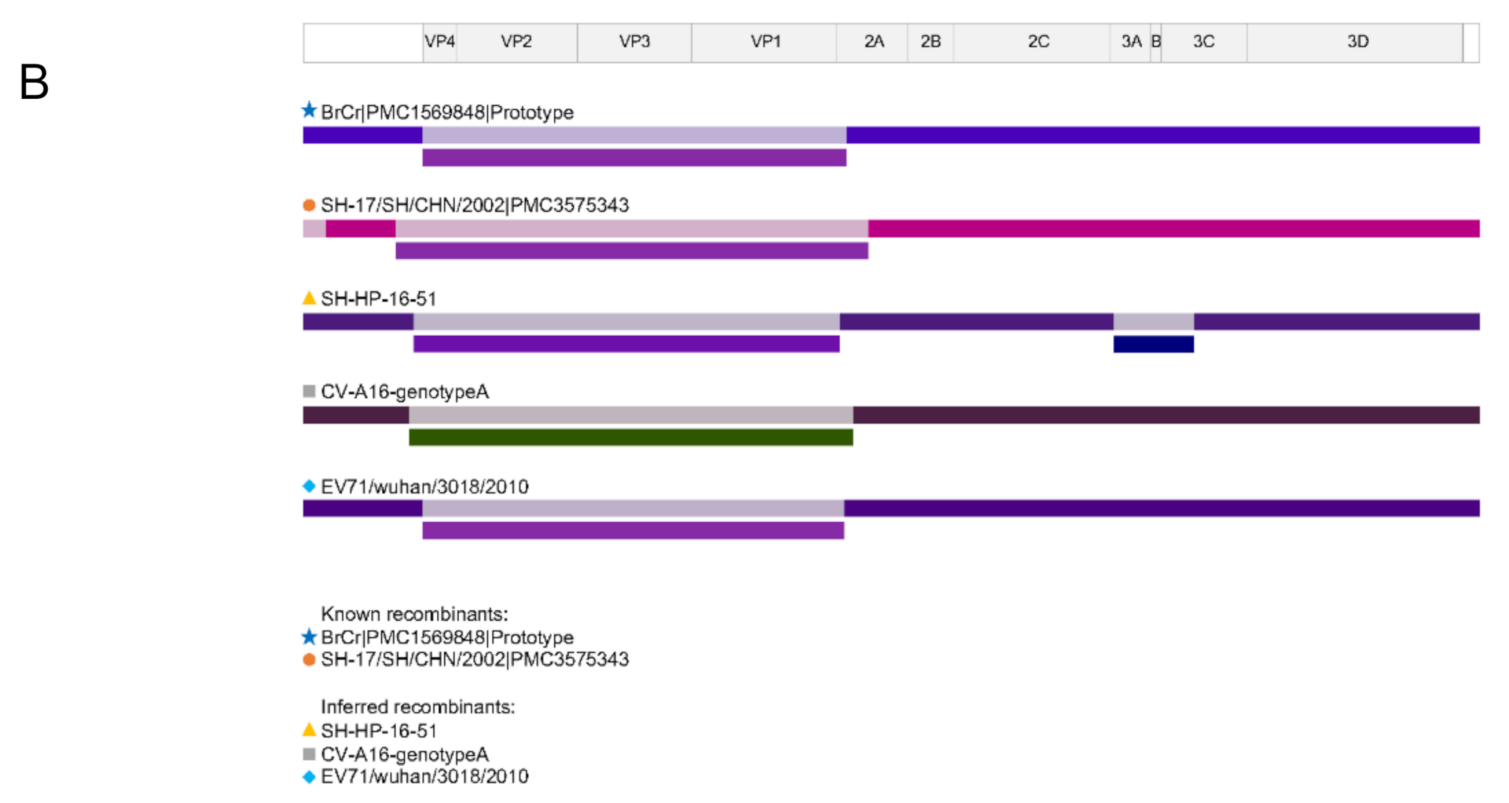
Recombination analysis of EV-A isolates. Gene sequences for the EV-A isolates selected in Figure 1 along with published recombinant strains BrCr (U22521) [136] and SH-17/SH/CHN/2002 (JX678885) [137] were analyzed. (**A**) Phylogenetic trees of different genomic regions. Individual gene sequences were retrieved from the ViPR website (https://www.viprbrc.org/). Trees were generated using RaXML with 100 bootstrap replicates, and subsequently visualized in Archaeopteryx.js on ViPR. CV-A16 strains are labeled in magenta; EV-A71 strains are labeled in green. Recombinant controls (BrCr, SH-17/SH/CHN/2002) are highlighted by navy and orange shapes, respectively. Three novel recombinants (SH-HP-16-51, CV-A16-genotypeA, EV71/wuhan/3018/2010) were inferred based on changes in their phylogenetic neighborhoods between individual genes and are highlighted by yellow, gray, and light blue shapes. (**B**) Recombination analysis of the same dataset using RDP4 [138] detected recombination events in recombinant controls (BrCr, SH-17/SH/CHN/2002) and the three inferred recombinants (SH-HP-16-51, CV-A16-genotypeA, EV71/wuhan/3018/2010) in regions consistent with the phylogenetic analysis.

A comparison of phylogenetics trees of protein sequences dispersed down the length of the genome can reveal sites of recombination (Figure 2A). With the VP1 protein serving as the reference tree, the CV-A16 (pink) and EV-A71 (green) isolates are clustered in the separate phylogenetic branches. In contrast, in the 2A and other non-structural protein trees, two EV-A71 isolates more closely cluster with CV-A16 isolates than with the other EV-A71s, suggesting a recombination event has occurred between a subset of EV-A71 and CV-A16 isolates somewhere between the VP1 and 2A genes. Recombination breakpoint analysis (Figure 2B) confirms these findings. Interestingly, in all five cases in which recombination between CV-A16 and EV-A71 has occurred, two breakpoints are observed, one between the 5′UTR and VP4 and the other between VP1 and 2A. Whether these hotspots relate to some special recombinogenic features of the EV-A viral genome or are due to selective pressure to keep the four capsid proteins together is unclear.

## 5. Enterovirus Surveillance and Sequence Coverage

Clinical enterovirus samples are collected in several countries around the world, mostly from voluntary opportunistic sampling. In the United States, the CDC collects enterovirus surveillance data and samples using two voluntary laboratory-based systems—the National Respiratory and Enteric Virus Surveillance System and the National Enterovirus Surveillance System (NESS). Because reporting is voluntary, the true prevalence of EV disease can be difficult to discern from these data. In addition to clinical surveillance, environmental surveillance is performed to a limited extent, usually from sewage samples to monitor polio eradication efforts [139]. Although far fewer samples are collected from environmental surveillance, it is nevertheless an important component of enterovirus surveillance systems for assessment of genetic diversity, first because asymptomatic or mild enterovirus infections would not be identified through clinical surveillance, and second for data in geographic regions where clinical surveillance is low. In contrast, some countries, such as Japan, have a sentinel-based surveillance system in which every patient with a defined set of symptoms is reported [31].

Some of these samples are subsequently sequenced and their sequences deposited in public databases. The Virus Pathogen Resource (ViPR; https://www.viprbrc.org) [140] was used to collect enterovirus sequence data and metadata to analyze the geographic and temporal patterns of enterovirus sequence coverage. The number of genomic sequence records for the most common non-polio EVs are shown in (Table 1). ViPR is part of the BV-BRC (Bacterial and Viral Bioinformatics Resource Center), a US National Institute of Allergy and Infectious Diseases (NIAID)-sponsored bioinformatics repository of data and analysis tools for major human viral pathogens, including enteroviruses [140]. ViPR collects data from several sources, including genome sequence data from NCBI GenBank and RefSeq. Virus type, sample origin, and sample collection date are supplied with each sequence, if available. As sequencing becomes more and more common and cost effective, the number of sequence records have increased considerably over the last two decades. While these data provide considerable insights into the evolution and spread of enteroviruses, there are significant gaps in current sequence coverage that should be noted. Isolates circulating in areas of low surveillance may be underreported. Even in regions with surveillance programs, geographic coverage is uneven due to voluntary reporting. To this end, 52% of current sequences from 2010 to 2018 are from the Asia–Pacific region, whereas only 3% and 1% are from Africa and South America, respectively; it is unlikely that these numbers represent true prevalence differences. Furthermore, there is a significant time delay in reporting sequences (often as much as 1–3 years after collection). Collection method differences may also lead to prevalence biases. For example, enterovirus surveillance studies generally collect stool samples that might result in the underreporting of some types, such as EV-D68, which tend to be absence from stool even during acute infection [17,115]. Virus isolates are also more likely to be represented in the sequence record whether they replicate in cell culture, are associated with an outbreak or pandemic, or cause severe disease. Some temporal patterns and geographic distributions of different disease causing EV types reflected by the sequence analysis described below do generally match the surveillance patterns reported in the literature. However, this may not reflect the actual distribution of these viral types and may instead reflect a lack of sequence coverage, emphasizing the need for more surveillance in underreported regions. Even with these collection biases in mind, some epidemiological patterns can still be observed in the sequence coverage.

## 6. Geographic Distribution of Enterovirus Sequences

**Figure 3:**
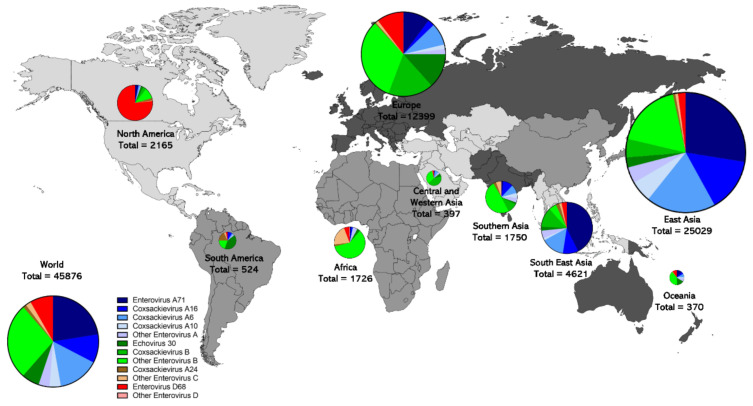
Geographic distribution of non-polio enterovirus genomic sequence records. The origin (if available) of all non-polio enterovirus A (blue) B (green) C (brown) and D (red) sequences from samples collected from 2000 to 2018 are shown, highlighting the different distribution patterns of enterovirus types across nine different regions of the world. Data were obtained using the ViPR database, as described in Table 1 footnotes. Regional pie graph sizes are proportional to the square root of total sequences of each group.

Different enterovirus species show different sequence coverage patterns across the world (Figure 3). Enterovirus A type sequences dominate in East Asia (71%) and Southeast Asia (73.4%). Generally, the EV-A types EV-A71, EV-A6, EV-A16, and EV-A10 are the most commonly sequenced types in East Asia, and Southeast Asia, in that order. There are low levels of EV-A coverage in Africa, North America, and Western Asia. Enterovirus B is sequenced most commonly in Western Asia (81.4%), Europe (63.1%), Africa (63.0%), South America (61.3%), Southern Asia (61.0%) and Oceania (55.1%). E30 represented 13.3% of sequences in Europe and 15.7% in Oceania. Interestingly, Europe has 57.4% of all E30 sequences. A significant proportion of the sequence coverage that exists in Africa (22.1%) and South America (21.2%) is EV-C, which is rarer in the rest of the world. CV-A24 is the most commonly sequenced EV-C type. In South America, a high proportion of the sequence coverage is CV-A24 (17.6%, 92 sequences) but there is a similar level of sequence coverage in South East Asia (166 sequences) and East Asia (103 sequences in absolute numbers), although the proportion is much lower. In Africa, other EV-C types represent a large share of sequence coverage (20.9%), Although this includes many types, the most common are EV-A13 (9.5%), EV-A20 (4.1%), and EV-C99 (3.1%). Enterovirus D sequences are relatively rare worldwide, except in North America, where they represent 76.7% of sequences, but Europe has similar sequence coverage (1283 vs. 1661 sequences in absolute numbers), although the proportion is lower (10.3%).

Differences in surveillance methodology undoubtedly contribute to this geographic pattern, as has already been discussed. For example, HFMD is a reportable disease in many countries in East Asia, such as Japan and China, leading to much higher collection patterns of viruses that cause this disease, also leading to much more sequencing of enteroviruses in East Asia. Indeed 54.6% of worldwide enterovirus sequences are from East Asia. Africa, Southern Asia, and Central and East Asia are undergoing poliovirus eradication efforts in which AFP is a reportable disease, and thus a large proportion of sequences in these regions are likely related to AFP. Climate and socio-economic factors likely contribute to the geographic distribution of different types [141]. Homotypic immunity that influences transmission dynamics may also contribute to the geographic distribution of types. The differences in surveillance undoubtably contribute to lower sequence coverage in geographic areas outside of East Asia and Europe, likely leading to underreporting.

## 7. Enterovirus Circulation and Temporal Dynamics

The long-term circulation dynamics of enteroviruses are characterized by two general patterns—endemic and epidemic [142]. An endemic pattern is observed when a type persists at a low level throughout the year and may or may not be detectable. An epidemic pattern is observed when expansions and contractions occur on an annual or multi-year cyclical pattern. New types or strains can start cryptically with an endemic pattern and then emerge as major epidemic strains, such as EV-A71 [143] and EV-D68 [144]. During the expansion/epidemic phase, diversity can increase significantly. For example, with EV-D68, multiple clades (A, B, and C) emerged within a 13 year period [144]. Interestingly, common population bottlenecks also seem to occur, as the most recent common ancestors of known isolates for each of the most common human EV types diverged only 55–200 years ago (typically less than 100 years), as evidenced by long phylogenetic branches leading from all contemporary isolates to the tree roots [10]. Severe bottlenecks seem to occur regularly from epidemic epidemiological cycles and may play a role in constraining diversity in certain geographic areas, suggesting a self-limiting pattern. One recent example is the possible extinction of E30 in Russia [145] and its decreased diversity in Europe and America [146,147]. Another example is the loss of clade A within EV-A71 [143].

**Figure 4:**
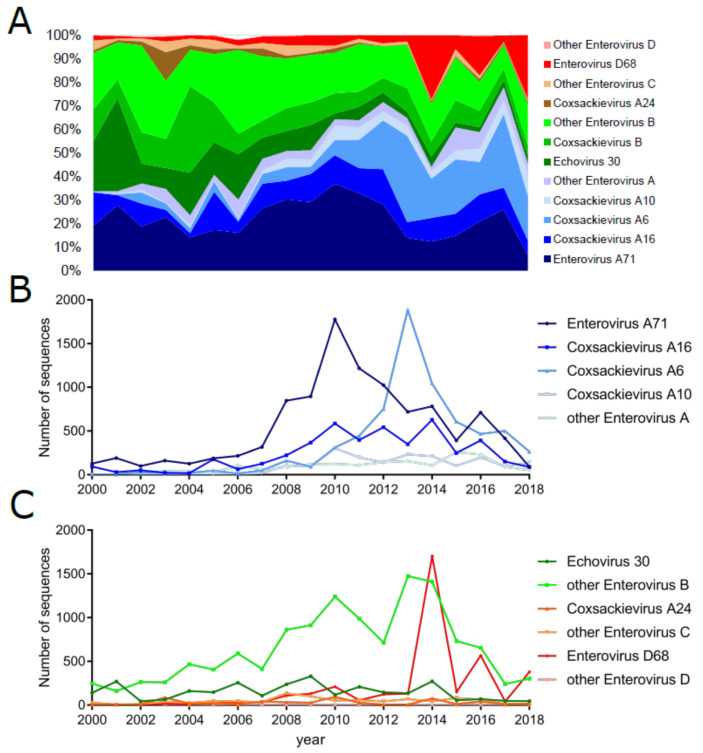
Enterovirus sequence representation from 2000 to 2018 by type. The sample collection calendar year (if available) of all non-polio enteroviruses A (blue), B (green), C (brown), and D (red) sequences. The percentage of each type collected during each year is given in panel (**A**). The numbers of sequences collected from the most common types of EV-A and EV-B, -C, and -D are displayed in panels (**B**) and (**C**), respectively. Data were obtained using the ViPR database, as described in Table 1 footnotes.

Changes between these circulation patterns affect the sequencing coverage of different EV types over time as different EV types become more or less prevalent (Figure 4). Due to severe outbreaks of HFMD in the Asia–Pacific region, China established a national enhanced surveillance system for HFMD in May of 2008 [19] and found that EV-A71, CV-A16, and CV-A6 are responsible for most HFMD cases. Indeed, the four most common types of enterovirus genome sequences in the ViPR database for isolates from the last five years (2014–2019) were EV-D68, EV-A71, CV-A6, and CV-A16, representing 62.6% of all sequences from that period (Table 1). EV-A71 peak collection occurred in 2010, CV-A6 in 2013 and EV-D68 in 2014, likely reflecting the initial emergence of significant outbreaks of each of these virus types (Figure 4A). For example, the large EV-D68 sequence coverage peak is reflective of a significant worldwide outbreak in 2014, primarily concentrated in North America [92,148,149,150], when viral samples were frequently sequenced. The 2002–2003 epidemic in Brazil of CV-A24 [151] can also be seen as a peak in coverage of CV-A24 sequences (Figure 4).

Depending on the geographic region, different types exhibit different circulation patterns. Epidemic enterovirus infections in temperate regions are typically characterized by a strong seasonal pattern, with climate-dependent peaks in the summer and early autumn. For example, in the United States, the mean timing of cases ranged from July in Texas to September in Colorado, with the dew point temperature explaining ∼30% of the variation in the intensity of transmission [152]. Northern China also shows similar seasonality patterns, experiencing a single annual peak in June, whereas southern China experiences less pronounced semi-annual outbreaks in May and September/October [19]. In general, more tropical regions (e.g., Florida) experience less pronounced seasonal peaks in transmission [152]. Differences in geography also can change the timing of epidemic cycles. For example, EV-A71 exhibits an annual cycle in China, peaking in June of each year [19,153]. However, in Malaysia [32,154] and Japan [155], EV-A71 exhibits a 3 year cyclic pattern. EV-D68 has recently emerged with a biennial circulation pattern [73,74]. The 2 year cycle of EV-D68 peak in prevalence can also be observed in sequence coverage (Figure 4), with an initial peak in 2014 and then others in 2016 and 2018, albeit at lower levels, likely due to fewer samples being sent for sequencing. Cyclic patterns can extend even longer, with CV-B4 exhibiting 4 year cycles [31]. Interestingly these cycles are not fixed—E30 has switched between 5 year and 3 year cycles [123,126,156,157,158]; and CV-A4 switched from a 1 year cycle to a 2 year cycle in 2004 [31]. These cyclical patterns are not fully understood and may be the result of numerous factors, including weather, geographic barriers, hygiene, viral evolution, herd immunity, changes in the susceptible population, and other host factors. A recent study using the Japanese sentinel-based surveillance and a stochastic transmission model based on demography [31] was able to effectively model the circulation dynamics of numerous EV types. The model assumed that births would replenish the susceptible pool and determined the chances of an outbreak occurring, demonstrating that acquired type-specific immunity could explain the epidemiological pattern of 18 of the 20 types analyzed. These findings support the idea that homotypic immunity is the most significant factor contributing to transmission dynamics [152]. The relative species prevalence of EV infection also changes throughout the year. For example, environmental sampling of sewage in Ohio in 2011 revealed that the composition of enterovirus species varied over the year with EV-B rising to 80% of sequences in the summer and fall and EV-A rising to 45% in the spring [9,159].

## 8. Conclusions/Perspective

In this review, we analyzed the complete collection of publicly available EV genomic sequences, including phylogenetic and recombination analysis, and evaluated the geographic and temporal patterns of sequence coverage of the different EV types. Sequence abundance appears correlated with outbreaks of EV types, both temporally and geographically, and epidemics can easily be identified in the sequence record. However, the lack of sequence reports in the developing world, and the lack of standardized collection and sequencing methods clearly skew the relationship between sequence representation and EV disease incidence, so it is clear that the sequence coverage does not represent true prevalence due to these sampling biases. This emphasizes the need for a coordinated global infectious disease research and surveillance strategy to better compare EV incidence across geographic regions. As discussed in this review, the composition of enterovirus cases has changed considerably throughout the world. The increasing prevalence of CV-A6 and CV-A10 in the Asia–Pacific region suggests the need for a HFMD vaccine strategy that includes those EV types, especially considering the incidence of severe HFMD attributed to CV-A6. The emergence of EV-D68, especially in North America, is implicated in a rise in AFM cases. In order to explore the causative relationship between EV-D68 and AFM, extended surveillance efforts, improved detection methods, and advanced molecular analysis of virulence determinants are needed. In addition, a potential role for virus-induced immune responses in neuropathology needs to be explored. While nearly every EV type has been identified in surveillance or case reports from AFP patients [17,56,57,160,161,162,163,164,165,166], EV-B-associated AFP deserves more attention. Because EV-B is such a diverse species and the frequency of outbreaks can have long cyclical patterns, it has been difficult to associate any specific EV-B type as a frequent cause of AFP. However, as a group, EV-B types have been identified with over 70% of the enterovirus-associated AFP cases.

In conclusion, although poliovirus and rhinovirus may be more widely recognized as human infectious disease agents, the non-polio and non-rhino enteroviruses are important global endemic and epidemic pathogens. Their wide genetic diversity makes it challenging to develop sensitive and specific diagnostic reagents or cross-protective preventative vaccines. The fact that enteroviruses can often cause severe disease in humans motivates their further study by the research community.

## Figures and Tables

**Table 1:** Number of genomic sequence records for the most common non-polio EVs.

Number of Sequences *			
All Time	2014 to 2019	Species	Type
7174	2890	A	Coxsackievirus A6
12720	2413	A	Enterovirus A71
5318	1627	A	Coxsackievirus A16
2068	742	A	Coxsackievirus A10
974	435	A	Coxsackievirus A4
418	139	A	Coxsackievirus A2
275	111	A	Coxsackievirus A5
2211	653	B	Echovirus 11
1850	612	B	Coxsackievirus B5
4402	516	B	Echovirus 30
2139	497	B	Echovirus 6
569	211	B	Echovirus 18
1244	194	B	Coxsackievirus B3
551	178	B	Echovirus 25
238	166	B	Echovirus 16
1100	129	B	Coxsackievirus B4
803	106	B	Echovirus 7
778	97	B	Echovirus 9
430	90	B	Echovirus 3
549	75	B	Coxsackievirus B2
539	69	B	Coxsackievirus B1
620	68	B	Coxsackievirus A9
105	2	B	Coxsackievirus B6
974	157	C	Coxsackievirus A24
258	33	C	Coxsackievirus A13
154	27	C	Coxsackievirus A21
133	27	C	Enterovirus C99
3841	2895	D	Enterovirus D68

* The number of sequence records were determined by searching for all human enteroviruses in ViPR on 9 October 2020, and aliases condensed by enterovirus type. The full table along with details on how the number of sequences was determined are shown in (Table S1).

## Supplementary Materials

The following are available online at https://www.mdpi.com/2076-2607/8/12/1856/s1, Table S1: Number of genomic sequence records for all non-polio EVs.

## References

[1] Pallansch M.A., Roos R.P., Knipe D.M., Howley P.M., Griffin D.E., Lamb R.A., Martin M.A., Roizman B., Stephen E.S. (2001). Enteroviruses: Polioviruses, coxsackieviruses, echoviruses, and newer enteroviruses. Fields Virology.

[2] Tangermann R.H., Aylward R., Birmingham M., Horner R., Olivé J.M., Nkowane B.M., Hull H.F., Burton A. (1997). Current status of the global eradication of poliomyelitis. World Health Stat. Q. Rapp. Trimest. Stat. Sanit. Mond..

[3] Shaghaghi M., Soleyman-Jahi S., Abolhassani H., Yazdani R., Azizi G., Rezaei N., Barbouche M.-R., McKinlay M.A., Aghamohammadi A. (2018). New insights into physiopathology of immunodeficiency-associated vaccine-derived poliovirus infection; systematic review of over 5 decades of data. Vaccine.

[4] Guo J., Bolivar-Wagers S., Srinivas N., Holubar M., Maldonado Y. (2015). Immunodeficiency-related vaccine-derived poliovirus (iVDPV) cases: A systematic review and implications for polio eradication. Vaccine.

[5] Burns C.C., Diop O.M., Sutter R.W., Kew O.M. (2014). Vaccine-Derived Polioviruses. J. Infect. Dis..

[6] Royston L., Tapparel C. (2016). Rhinoviruses and Respiratory Enteroviruses: Not as Simple as ABC. Viruses.

[7] Drysdale S.B., Mejias A., Ramilo O. (2017). Rhinovirus—Not just the common cold. J. Infect..

[8] (ICTV) ICoToV (2019). Virus Taxonomy: 2019 Release. https://talk.ictvonline.org/taxonomy/.

[9] Oberste M.S., Maher K., Kilpatrick D.R., Pallansch M.A. (1999). Molecular Evolution of the Human Enteroviruses: Correlation of Serotype with VP1 Sequence and Application to Picornavirus Classification. J. Virol..

[10] Lukashev A.N., Vakulenko Y.A. (2017). Molecular evolution of types in non-polio enteroviruses. J. Gen. Virol..

[11] Zhang Y., Cao J., Zhang S., Lee A.J., Sun G., Larsen C.N., Zhao H., Gu Z., He S., Klem E.B. (2016). Genetic changes found in a distinct clade of Enterovirus D68 associated with paralysis during the 2014 outbreak. Virus Evol..

[12] Pallansch M.A., Sandhu H.S. (2006). The Eradication of Polio—Progress and Challenges. N. Engl. J. Med..

[13] Witsø E., Palacios G., Cinek O., Stene L.C., Grinde B., Janowitz D., Lipkin W.I., Rønningen K.S. (2006). High Prevalence of Human Enterovirus A Infections in Natural Circulation of Human Enteroviruses. J. Clin. Microbiol..

[14] Ma E., Chan K.C., Cheng P., Wong C., Chuang S.K. (2010). The enterovirus 71 epidemic in 2008—Public health implications for Hong Kong. Int. J. Infect. Dis..

[15] Tapparel C., Siegrist F., Petty T.J., Kaiser L. (2013). Picornavirus and enterovirus diversity with associated human diseases. Infect. Genet. Evol..

[16] Klein M., Chong P. (2015). Is a multivalent hand, foot, and mouth disease vaccine feasible?. Hum. Vaccines Immunother..

[17] Suresh S., Forgie S., Robinson J. (2018). Non-polio Enterovirus detection with acute flaccid paralysis: A systematic review. J. Med. Virol..

[18] Liu J., Xiang X., Pu Z., Long Y., Xiao D., Zhang W., Li Q., Li X., Li S., Shao Z. (2019). Epidemic pattern of hand-foot-and-mouth disease in Xi’an, China from 2008 through 2015. BMC Infect. Dis..

[19] Xing W., Liao Q., Viboud C., Zhang J., Sun J. (2014). Epidemiological characteristics of hand-foot-and-mouth disease in China, 2008–2012. Lancet Infect. Dis..

[20] Shi L., Zhao H., Wu D. (2018). Modelling and analysis of HFMD with the effects of vaccination, contaminated environments and quarantine in mainland China. Math. Biosci. Eng. MBE.

[21] Takahashi S., Metcalf C.J.E., Arima Y., Fujimoto T., Shimizu H., Van Doorn H.R., Le Van T., Chan Y.-F., Farrar J.J., Oishi K. (2018). Epidemic dynamics, interactions and predictability of enteroviruses associated with hand, foot and mouth disease in Japan. J. R. Soc. Interface.

[22] Huang W., Huang L., Lu C., Cheng A.-L., Chang L.-Y. (2013). Atypical hand-foot-mouth disease in children: A hospital-based prospective cohort study. Virol. J..

[23] Chua K., Kasri A. (2011). Hand foot and mouth disease due to enterovirus 71 in Malaysia. Virol. Sin..

[24] Ang L.W., Koh B.K., Chan K.P., Chua L.T., James L., Goh K.T. (2009). Epidemiology and control of hand, foot and mouth disease in Singapore, 2001–2007. Ann. Acad. Med. Singap..

[25] Nhan L.N.T., Hong N.T.T., Nhu L.N.T., Nguyet L.A., Ny N.T.H., Thanh T.T., Han D.D.K., Van H.M.T., Thwaites C.L., Hien T.T. (2018). Severe enterovirus A71 associated hand, foot and mouth disease, Vietnam, 2018: Preliminary report of an impending outbreak. Eurosurveillance.

[26] Anh N.T., Nhu L.N.T., Van H.M.T., Hong N.T.T., Thanh T.T., Hang V.T.T., Ny N.T.H., Nguyet L.A., Phuong T.T.L., Nhan L.N.T. (2018). Emerging Coxsackievirus A6 Causing Hand, Foot and Mouth Disease, Vietnam. Emerg. Infect. Dis..

[27] Hoang M.T.V., Nguyen T.A., Tran T.T., Vu T.T.H., Le N.T.N., Nguyen T.H.N., Le T.H.N., Nguyen H.T.T., Nguyen T.H., Le N.T.N. (2019). Clinical and aetiological study of hand, foot and mouth disease in southern Vietnam, 2013–2015: Inpatients and outpatients. Int. J. Infect. Dis..

[28] Noisumdaeng P., Korkusol A., Prasertsopon J., Sangsiriwut K., Chokephaibulkit K., Mungaomklang A., Thitithanyanont A., Buathong R., Guntapong R., Puthavathana P. (2019). Longitudinal study on enterovirus A71 and coxsackievirus A16 genotype/subgenotype replacements in hand, foot and mouth disease patients in Thailand, 2000–2017. Int. J. Infect. Dis..

[29] Puenpa J., Auphimai C., Korkong S., Vongpunsawad S., Poovorawan Y. (2018). Enterovirus A71 Infection, Thailand, 2017. Emerg. Infect. Dis..

[30] Duong V., Mey C., Eloit M., Zhu H., Danet L., Huang Z., Zou G., Tarantola A., Cheval J., Perot P. (2016). Molecular epidemiology of human enterovirus 71 at the origin of an epidemic of fatal hand, foot and mouth disease cases in Cambodia. Emerg. Microbes Infect..

[31] Pons-Salort M., Grassly N.C. (2018). Serotype-specific immunity explains the incidence of diseases caused by human enteroviruses. Science.

[32] NikNadia N., Sam I.C., Rampal S., WanNorAmalina W., NurAtifah G., Verasahib K., Ong C.C., MohdAdib M., Chan Y.F. (2016). Cyclical Patterns of Hand, Foot and Mouth Disease Caused by Enterovirus A71 in Malaysia. PLoS Negl. Trop. Dis..

[33] Chen L., Yao X.J., Xu S.J., Yang H., Wu C.L., Lu J., Xu W.-B., Zhang H., Meng J., Zhang Y. (2019). Molecular surveillance of coxsackievirus A16 reveals the emergence of a new clade in mainland China. Arch. Virol..

[34] Ji T., Guo Y., Huang W., Shi Y., Xu Y., Tong W., Yao W., Tan Z., Zeng H., Ma J. (2018). The emerging sub-genotype C_2_ of CoxsackievirusA10 Associated with Hand, Foot and Mouth Disease extensively circulating in mainland of China. Sci. Rep..

[35] Puenpa J., Vongpunsawad S., Österback R., Waris M., Eriksson E., Albert J., Midgley S., Fischer T.K., Eis-Hübinger A.M., Cabrerizo M. (2016). Molecular epidemiology and the evolution of human coxsackievirus A6. J. Gen. Virol..

[36] Wang S.H., Wang A., Liu P.P., Zhang W.Y., Du J., Xu S., Liu G.-C., Zheng B.-S., Huan C., Zhao K. (2018). Divergent Pathogenic Properties of Circulating Coxsackievirus A6 Associated with Emerging Hand, Foot, and Mouth Disease. J. Virol..

[37] Bian L., Wang Y., Yao X., Mao Q., Xu M., Liang Z. (2015). Coxsackievirus A6: A new emerging pathogen causing hand, foot and mouth disease outbreaks worldwide. Expert Rev. Anti Infect. Ther..

[38] Song Y., Zhang Y., Ji T., Gu X., Yang Q., Zhu S., Xu W., Xu Y., Shi Y., Huang X. (2017). Persistent circulation of Coxsackievirus A6 of genotype D3 in mainland of China between 2008 and 2015. Sci. Rep..

[39] Huang J., Liao Q., Ooi M.H., Cowling B.J., Chang Z., Wu P., Liu F., Li Y., Luo L., Yu S. (2018). Epidemiology of Recurrent Hand, Foot and Mouth Disease, China, 2008–2015. Emerg. Infect. Dis..

[40] Bessaud M., Razafindratsimandresy R., Nougairède A., Joffret M.L., Deshpande J.M., Dubot-Pérès A., Héraud J.-M., De Lamballerie X., Delpeyroux F., Bailly J.-L. (2014). Molecular Comparison and Evolutionary Analyses of VP1 Nucleotide Sequences of New African Human Enterovirus 71 Isolates Reveal a Wide Genetic Diversity. PLoS ONE.

[41] Wang J., Teng Z., Cui X., Li C., Pan H., Zheng Y., Mao S., Yang Y., Wu L., Guo X. (2018). Epidemiological and serological surveillance of hand-foot-and-mouth disease in Shanghai, China, 2012–2016. Emerg. Microbes Infect..

[42] Li J., Pan H., Wang X., Zhu Q., Ge Y., Cai J., Li Y., Xia A., Hu J., Zeng M. (2018). Epidemiological surveillance of hand, foot and mouth disease in Shanghai in 2014–2016, prior to the introduction of the enterovirus 71 vaccine. Emerg. Microbes Infect..

[43] Wang J., Hu T., Sun D., Ding S., Carr M.J., Xing W., Li S., Wang X., Shi W. (2017). Epidemiological characteristics of hand, foot, and mouth disease in Shandong, China, 2009–2016. Sci. Rep..

[44] Ooi M.H., Wong S.C., Lewthwaite P., Cardosa M.J., Solomon T. (2010). Clinical features, diagnosis, and management of enterovirus 71. Lancet Neurol..

[45] Teoh H.L., Mohammad S.S., Britton P.N., Kandula T., Lorentzos M.S., Booy R., Jones C.A., Rawlinson W., Ramachandran V., Rodriguez M.L. (2016). Clinical Characteristics and Functional Motor Outcomes of Enterovirus 71 Neurological Disease in Children. JAMA Neurol..

[46] Wang S.-M., Liu C.C., Tseng H.W., Wang J.-R., Huang C.-C., Chen Y.-J., Yang Y., Lin S., Yeh T. (1999). Clinical Spectrum of Enterovirus 71 Infection in Children in Southern Taiwan, with an Emphasis on Neurological Complications. Clin. Infect. Dis..

[47] McMinn P., Stratov I., Nagarajan L., Davis S. (2001). Neurological Manifestations of Enterovirus 71 Infection in Children during an Outbreak of Hand, Foot, and Mouth Disease in Western Australia. Clin. Infect. Dis..

[48] Pérez-Vélez C.M., Anderson M.S., Robinson C.C., McFarland E.J., Nix W.A., Pallansch M.A., Oberste M.S., Glodé M.P. (2007). Outbreak of Neurologic Enterovirus Type 71 Disease: A Diagnostic Challenge. Clin. Infect. Dis..

[49] Chen C.Y., Chang Y.C., Huang C.C., Lui C.C., Lee K.W., Huang S.C. (2001). Acute flaccid paralysis in infants and young children with enterovirus 71 infection: MR imaging findings and clinical correlates. Am. J. Neuroradiol..

[50] Schubert R.D., Hawes I.A., Ramachandran P.S., Ramesh A., Crawford E.D., Pak J.E., Wu W., Cheung C.K., O’Donovan B.D., Tato C.M. (2019). Pan-viral serology implicates enteroviruses in acute flaccid myelitis. Nat. Med..

[51] Yu P., Bao L., Xu L., Li F., Lv Q., Deng W., Xu Y., Qin C. (2017). Neurotropism In Vitro and Mouse Models of Severe and Mild Infection with Clinical Strains of Enterovirus 71. Viruses.

[52] Lin J., Shih S. (2014). Cell and tissue tropism of enterovirus 71 and other enteroviruses infections. J. Biomed. Sci..

[53] Mandary M.B., Poh C.L. (2018). Changes in the EV-A71 Genome through Recombination and Spontaneous Mutations: Impact on Virulence. Viruses.

[54] Mao Q., Wang Y., Bian L., Xu M., Liang Z. (2016). EV-A71 vaccine licensure: A first step for multivalent enterovirus vaccine to control HFMD and other severe diseases. Emerg. Microbes Infect..

[55] Yee P.T.I., Poh C.L. (2017). Impact of genetic changes, pathogenicity and antigenicity on Enterovirus- A71 vaccine development. Virology.

[56] Tao Z., Wang H., Liu Y., Li Y., Jiang P., Liu G., Lin X., Li M., Wang S., Ji F. (2014). Non-Polio Enteroviruses from Acute Flaccid Paralysis Surveillance in Shandong Province, China, 1988–2013. Sci. Rep..

[57] Masa-Calles J., Torner N., López-Perea N., Torres de Mier M.V., Fernández-Martínez B., Cabrerizo M., Gallardo-García V., Malo C., Margolles-Martins M.J., Portell M. (2018). Acute flaccid paralysis (AFP) surveillance: Challenges and opportunities from 18 years’ experience, Spain, 1998 to 2015. Eurosurveillance.

[58] Fernandez-Garcia M.D., Kebe O., Fall A.D., Ndiaye K. (2017). Identification and molecular characterization of non-polio enteroviruses from children with acute flaccid paralysis in West Africa, 2013–2014. Sci. Rep..

[59] Delogu R., Battistone A., Buttinelli G., Fiore S., Fontana S., Amato C., Cristiano K., Gamper S., Simeoni J., Frate R. (2018). Poliovirus and Other Enteroviruses from Environmental Surveillance in Italy, 2009–2015. Food Environ. Virol..

[60] Verma N.A., Zheng X.T., Harris M.U., Cadichon S.B., Melin-Aldana H., Khetsuriani N., Oberste M.S., Shulman S.T. (2009). Outbreak of Life-Threatening Coxsackievirus B1 Myocarditis in Neonates. Clin. Infect. Dis..

[61] Garmaroudi F.S., Marchant D., Hendry R., Luo H., Yang D., Ye X., Shi J., McManus B.M. (2015). Coxsackievirus B3 replication and pathogenesis. Future Microbiol..

[62] Schmidt N.J., Magoffin R.L., Lennette E.H. (1973). Association of group B coxsackie viruses with cases of pericarditis, myocarditis, or pleurodynia by demonstration of immunoglobulin M antibody. Infect. Immun..

[63] Yin-Murphy M., Lim K.H., Ho Y.M. (1976). A coxsackievirus type A24 epidemic of acute conjunctivitis. Southeast Asian J. Trop. Med. Public Health.

[64] Tavares F., Campos R.D.M., Burlandy F., Fontella R., De Melo M.M.M., Da Costa E.V., Da Silva E.E. (2011). Molecular Characterization and Phylogenetic Study of Coxsackievirus A24v Causing Outbreaks of Acute Hemorrhagic Conjunctivitis (AHC) in Brazil. PLoS ONE.

[65] Medina N.H., Haro-Munoz E., Pellini A.C., Machado B.C., Russo D.H., Timenetsky M.D., Carmona R.D.C.C. (2016). Acute hemorrhagic conjunctivitis epidemic in Sao Paulo State, Brazil, 2011. Rev. Panam. Salud Publica.

[66] Zhang Y., Sun Q., Cui H., Yan D., Fan Q., Song Y., Zhu S., Li X., Huang G., Ji T. (2016). Circulation of multiple serotypes of highly divergent enterovirus C in the Xinjiang Uighur Autonomous Region of China. Sci. Rep..

[67] Enfissi A., Joffret M.L., Delaune D., Delpeyroux F., Rousset D., Bessaud M. (2017). Coxsackievirus A24 Variant Associated with Acute Haemorrhagic Conjunctivitis Cases, French Guiana, 2017. Intervirology.

[68] Shukla D., Kumar A., Srivastava S., Dhole T.N. (2013). Molecular identification and phylogenetic study of coxsackievirus A24 variant isolated from an outbreak of acute hemorrhagic conjunctivitis in India in 2010. Arch. Virol..

[69] Burr S.E., Sillah A., Joof H., Bailey R.L., Holland M.J. (2017). An outbreak of acute haemorrhagic conjunctivitis associated with coxsackievirus A24 variant in The Gambia, West Africa. BMC Res. Notes.

[70] Zhang L., Zhao N., Huang X., Jin X., Geng X., Chan T.C., Liu S.-L. (2017). Molecular epidemiology of acute hemorrhagic conjunctivitis caused by coxsackie A type 24 variant in China, 2004–2014. Sci. Rep..

[71] Schieble J.H., Fox V.L., Lennette E.H. (1967). A probable new human picornavirus associated with respiratory diseases. Am. J. Epidemiol..

[72] Imamura T., Oshitani H. (2015). Global reemergence of enterovirus D68 as an important pathogen for acute respiratory infections. Rev. Med. Virol..

[73] Holm-Hansen C.C., Midgley S.E., Fischer T.K. (2016). Global emergence of enterovirus D68: A systematic review. Lancet Infect. Dis..

[74] Kramer R., Sabatier M., Wirth T., Pichon M., Lina B., Schuffenecker I., Josset L. (2018). Molecular diversity and biennial circulation of enterovirus D68: A systematic screening study in Lyon, France, 2010 to 2016. Eurosurveillance.

[75] Bowers J.R., Valentine M., Harrison V., Fofanov V.Y., Gillece J., Delisle J., Patton B., Schupp J., Sheridan K., Lemmer D. (2019). Genomic Analyses of Acute Flaccid Myelitis Cases among a Cluster in Arizona Provide Further Evidence of Enterovirus D68 Role. mBio.

[76] Force TUKAFP (2019). An increase in reports of acute flaccid paralysis (AFP) in the United Kingdom, 1 January 2018–21 January 2019: Early findings. Eurosurveillance.

[77] Cottrell S., Moore C., Perry M., Hilvers E., Williams C., Shankar A.G. (2018). Prospective enterovirus D68 (EV-D68) surveillance from September 2015 to November 2018 indicates a current wave of activity in Wales. Eurosurveillance.

[78] Aliabadi N., Messacar K., Pastula D.M., Robinson C.C., Leshem E., Sejvar J.J., Nix W.A., Oberste M.S., Feikin D.R., Dominguez S.R. (2016). Enterovirus D68 Infection in Children with Acute Flaccid Myelitis, Colorado, USA, 2014. Emerg. Infect. Dis..

[79] McKay S.L., Lee A.D., Lopez A.S., Nix W.A., Dooling K.L., Keaton A.A., Spence-Davizon E., Herlihy R., Clark T.A., Hopkins S.E. (2018). Increase in Acute Flaccid Myelitis—United States, 2018. MMWR Morb. Mortal. Wkly. Rep..

[80] Harrison C.J., Weldon W.C., Pahud B.A., Jackson M.A., Oberste M.S., Selvarangan R. (2019). Neutralizing Antibody against Enterovirus D68 in Children and Adults before 2014 Outbreak, Kansas City, Missouri, USA(1). Emerg. Infect. Dis..

[81] Brown D.M., Hixon A.M., Oldfield L.M., Zhang Y., Novotny M., Wang W., Das S.R., Shabman R.S., Tyler K.L., Scheuermann R.H. (2018). Contemporary Circulating Enterovirus D68 Strains Have Acquired the Capacity for Viral Entry and Replication in Human Neuronal Cells. mBio.

[82] Carballo C.M., Erro M.G., Sordelli N., Vazquez G., Mistchenko A.S., Cejas C., Rodriguez M., Cisterna D.M., Freire M.C., Contrini M.M. (2019). Acute Flaccid Myelitis Associated with Enterovirus D68 in Children, Argentina, 2016. Emerg. Infect. Dis..

[83] Knoester M., Helfferich J., Poelman R., Van Leer-Buter C., Brouwer O.F., Niesters H.G.M. (2019). Twenty-nine Cases of Enterovirus-D68–associated Acute Flaccid Myelitis in Europe 2016: A Case Series and Epidemiologic Overview. Pediatr. Infect. Dis. J..

[84] Bal A., Sabatier M., Wirth T., Coste-Burel M., Lazrek M., Stefic K., Brengel-Pesce K., Morfin F., Lina B., Schuffenecker I. (2019). Emergence of enterovirus D68 clade D1, France, August to November 2018. Eurosurveillance.

[85] Pellegrinelli L., Giardina F., Lunghi G., Renteria S.C., Greco L., Fratini A., Galli C., Piralla A., Binda S., Pariani E. (2019). Emergence of divergent enterovirus (EV) D68 sub-clade D1 strains, northern Italy, September to October 2018. Eurosurveillance.

[86] Kaida A., Iritani N., Yamamoto S.P., Kanbayashi D., Hirai Y., Togawa M., Amo K., Kohdera U., Nishigaki T., Shiomi M. (2017). Distinct genetic clades of enterovirus D68 detected in 2010, 2013, and 2015 in Osaka City, Japan. PLoS ONE.

[87] Sejvar J.J., Lopez A.S., Cortese M.M., Leshem E., Pastula D.M., Miller L., Glaser C., Kambhampati A., Shioda K., Aliabadi N. (2016). Acute Flaccid Myelitis in the United States, August–December 2014: Results of Nationwide Surveillance. Clin. Infect. Dis..

[88] Messacar K., Schreiner T.L., Maloney A.J., Wallace A., Ludke J., Oberste M.S., Nix W.A., Robinson C.C., Glodé M.P., Abzug M.J. (2015). A cluster of acute flaccid paralysis and cranial nerve dysfunction temporally associated with an outbreak of enterovirus D68 in children in Colorado, USA. Lancet.

[89] Van Haren K., Ayscue P., Waubant E., Clayton A., Sheriff H., Yagi S., Glenn-Finer R., Padilla T., Strober J.B., Aldrovandi G.M. (2015). Acute Flaccid Myelitis of Unknown Etiology in California, 2012–2015. JAMA.

[90] Ayscue P., Van Haren K., Sheriff H., Waubant E., Waldron P., Yagi S., Yen C., Clayton A., Padilla T., Pan C. (2014). Acute Flaccid Paralysis with Anterior Myelitis—California, June 2012–June 2014. MMWR Morb. Mortal. Wkly. Rep..

[91] Greninger A.L., Naccache S.N., Messacar K., Clayton A., Yu G., Somasekar S., Federman S., Stryke D., Anderson C., Yagi S. (2015). A novel outbreak enterovirus D68 strain associated with acute flaccid myelitis cases in the USA (2012–2014): A retrospective cohort study. Lancet Infect. Dis..

[92] Messacar K., Schreiner T.L., Van Haren K., Yang M., Glaser C.A., Tyler K.L., Dominguez S.R. (2016). Acute flaccid myelitis: A clinical review of US cases 2012–2015. Ann. Neurol..

[93] Messacar K., Abzug M.J., Dominguez S.R. (2016). 2014 outbreak of enterovirus D68 in North America. J. Med. Virol..

[94] Ng T.F., Montmayeur A., Castro C., Cone M., Stringer J., Lamson D.M., Rogers S.L., Chern S.-W.W., Magaña L., Marine R.L. (2016). Detection and Genomic Characterization of Enterovirus D68 in Respiratory Samples Isolated in the United States in 2016. Genome Announc..

[95] Lang M., Mirand A., Savy N., Henquell C., Maridet S., Perignon R., Labbé A., Peigue-Lafeuille H. (2014). Acute flaccid paralysis following enterovirus D68 associated pneumonia, France, 2014. Eurosurveillance.

[96] Bragstad K., Jakobsen K., Rojahn A.E., Skram M.K., Vainio K., Holberg-Petersen M., Hungnes O., Dudman S.G., Kran A.-M.B. (2014). High frequency of enterovirus D68 in children hospitalised with respiratory illness in Norway, autumn 2014. Influ. Other Respir. Viruses.

[97] Levy A., Roberts J.A., Lang J., Tempone S., Kesson A., Dofai A., Daley A.J., Thorley B.R., Speers D.J. (2015). Enterovirus D68 disease and molecular epidemiology in Australia. J. Clin. Virol..

[98] Dyda A., Stelzer-Braid S., Adam D., Chughtai A.A., MacIntyre C.R. (2018). The association between acute flaccid myelitis (AFM) and Enterovirus D68 (EV-D68)—What is the evidence for causation?. Eurosurveillance.

[99] Chong P.F., Kira R., Mori H., Okumura A., Torisu H., Yasumoto S., Shimizu H., Fujimoto T., Hanaoka N., Kusunoki S. (2018). Clinical Features of Acute Flaccid Myelitis Temporally Associated With an Enterovirus D68 Outbreak: Results of a Nationwide Survey of Acute Flaccid Paralysis in Japan, August–December 2015. Clin. Infect. Dis..

[100] Xiang Z., Xie Z., Liu L., Ren L., Xiao Y., Paranhos-Baccalà G., Wang J. (2016). Genetic divergence of enterovirus D68 in China and the United States. Sci. Rep..

[101] Zhang T.G., Li H.Q., Li A.H., Chen M., Gong C., Luo M., Dong M., Huang F. (2016). The Genomic Characterization of Enterovirus D68 from 2011 to 2015 in Beijing, China. Biomed. Environ. Sci..

[102] Zhang C., Zhang X., Dai W., Liu Q., Xiong P., Wang S., Geng L., Gong S., Huang Z. (2018). A Mouse Model of Enterovirus D68 Infection for Assessment of the Efficacy of Inactivated Vaccine. Viruses.

[103] Hixon A.M., Yu G., Leser J.S., Yagi S., Clarke P., Chiu C.Y., Tyler K.L. (2017). A mouse model of paralytic myelitis caused by enterovirus D68. PLoS Pathog..

[104] Tan Y., Hassan F., Schuster J.E., Simenauer A., Selvarangan R., Halpin R.A., Lin X., Fedorova N., Stockwell T.B., Lam T.T.-Y. (2015). Molecular Evolution and Intraclade Recombination of Enterovirus D68 during the 2014 Outbreak in the United States. J. Virol..

[105] Gong Y.N., Yang S.L., Shih S.R., Huang Y.C., Chang P.Y., Huang C.G., Kao K.-C., Hu H.-C., Liu Y.-C., Tsao K.-C. (2016). Molecular evolution and the global reemergence of enterovirus D68 by genome-wide analysis. Medicine (Baltim.).

[106] Yip C.C.Y., Lo J.Y.C., Sridhar S., Lung D.C., Luk S., Chan K.-H., Chan J.F.W., Cheng V.C.C., Woo P.C.Y., Yuen K.-Y. (2017). First Report of a Fatal Case Associated with EV-D68 Infection in Hong Kong and Emergence of an Interclade Recombinant in China Revealed by Genome Analysis. Int. J. Mol. Sci..

[107] Lau S.K., Yip C.C., Zhao P.S., Chow W.N., To K.K., Wu A.K., Yuen K.-Y., Woo P.C.Y. (2016). Enterovirus D68 Infections Associated with Severe Respiratory Illness in Elderly Patients and Emergence of a Novel Clade in Hong Kong. Sci. Rep..

[108] Dyrdak R., Grabbe M., Hammas B., Ekwall J., Hansson E.K., Luthander J., Naucler P., Reinius H., Rotzén-Östlund M., Albert J. (2016). Outbreak of enterovirus D68 of the new B3 lineage in Stockholm, Sweden, August to September 2016. Eurosurveillance.

[109] Knoester M., Schölvinck E.H., Poelman R., Smit S., Vermont C.L., Niesters H.G., Van Leer-Buter C.C. (2017). Upsurge of Enterovirus D68, the Netherlands, 2016. Emerg. Infect. Dis..

[110] Esposito S., Chidini G., Cinnante C., Napolitano L., Giannini A., Terranova L., Niesters H.G.M., Principi N., Calderini E. (2017). Acute flaccid myelitis associated with enterovirus-D68 infection in an otherwise healthy child. Virol. J..

[111] Wang G., Zhuge J., Huang W., Nolan S.M., Gilrane V.L., Yin C., Dimitrova N., Fallon J.T. (2017). Enterovirus D68 Subclade B3 Strain Circulating and Causing an Outbreak in the United States in 2016. Sci. Rep..

[112] Huang W., Wang G., Zhuge J., Nolan S.M., Dimitrova N., Fallon J.T. (2015). Whole-Genome Sequence Analysis Reveals the Enterovirus D68 Isolates during the United States 2014 Outbreak Mainly Belong to a Novel Clade. Sci. Rep..

[113] Bitnun A., Yeh E.A. (2018). Acute Flaccid Paralysis and Enteroviral Infections. Curr. Infect. Dis. Rep..

[114] Midgley C., Jackson M.A., Selvarangan R., Turabelidze G., Obringer E., Johnson D., Giles B.L., Patel A., Echols F., Oberste M.S. (2014). Severe Respiratory Illness Associated with Enterovirus D68—Missouri and Illinois, 2014. MMWR Morb. Mortal. Wkly. Rep..

[115] Harvala H., Broberg E., Benschop K., Berginc N., Ladhani S.N., Susi P., Christiansen C., McKenna J., Allen D., Makiello P. (2018). Recommendations for enterovirus diagnostics and characterisation within and beyond Europe. J. Clin. Virol..

[116] Savolainen-Kopra C., Blomqvist S. (2010). Mechanisms of genetic variation in polioviruses. Rev. Med. Virol..

[117] Sanjuán R. (2012). From Molecular Genetics to Phylodynamics: Evolutionary Relevance of Mutation Rates Across Viruses. PLoS Pathog..

[118] Zhang Y., Wang J., Guo W., Wang H., Zhu S., Wang D., Bai R., Li X., Yan D., Wang H. (2011). Emergence and Transmission Pathways of Rapidly Evolving Evolutionary Branch C4a Strains of Human Enterovirus 71 in the Central Plain of China. PLoS ONE.

[119] Liu W., Wu S., Xiong Y., Li T., Wen Z., Yan M., Qin K., Liu Y., Wu J. (2014). Co-Circulation and Genomic Recombination of Coxsackievirus A16 and Enterovirus 71 during a Large Outbreak of Hand, Foot, and Mouth Disease in Central China. PLoS ONE.

[120] Zhang Y., Zhu Z., Yang W., Ren J., Tan X., Wang Y., Naiying M., Xu S., Zhu S., Cui A. (2010). An emerging recombinant human enterovirus 71 responsible for the 2008 outbreak of Hand Foot and Mouth Disease in Fuyang city of China. Virol. J..

[121] Chen S.-P., Huang Y.-C., Li W.-C., Chiu C.-H., Huang C.-G., Tsao K.-C., Lin T.-Y. (2010). Comparison of Clinical Features Between Coxsackievirus A2 and Enterovirus 71 During the Enterovirus Outbreak in Taiwan, 2008: A Children’s Hospital Experience. J. Microbiol. Immunol. Infect..

[122] Lu Q.-B., Zhang X.-A., Wo Y., Xu H.-M., Li X.-J., Wang X.-J., Ding S.-J., Chen X.-D., He C., Liu L.-J. (2012). Circulation of Coxsackievirus A10 and A6 in Hand-Foot-Mouth Disease in China, 2009–2011. PLoS ONE.

[123] Leitch E.C.M., Bendig J., Cabrerizo M., Cardosa J., Hyypiä T., Ivanova O.E., Kelly A., Kroes A.C.M., Lukashev A., Macadam A. (2009). Transmission Networks and Population Turnover of Echovirus 30. J. Virol..

[124] Machado R.S., Ferreira J.L., Alves J.C.S., Bandeira R.S., Lemos P.S., Filho L.C.F., Cardoso J.F., Vianez-Junior J.L.S.G., Tavares F.N. (2019). Nearly Complete Genome Sequences of Enterovirus 96 and Enterovirus 99 Strains Isolated in the Northern Region of Brazil. Microbiol. Resour. Announc..

[125] Zhang Y., Tan X., Cui A., Mao N., Xu S., Zhu Z., Zhou J., Shi J., Zhao Y., Wang X. (2013). Complete Genome Analysis of the C4 Subgenotype Strains of Enterovirus 71: Predominant Recombination C4 Viruses Persistently Circulating in China for 14 Years. PLoS ONE.

[126] Leitch E.C.M., Cabrerizo M., Cardosa J., Harvala H., Ivanova O.E., Kroes A.C.M., Lukashev A., Muir P., Odoom J., Roivainen M. (2010). Evolutionary Dynamics and Temporal/Geographical Correlates of Recombination in the Human Enterovirus Echovirus Types 9, 11, and 30. J. Virol..

[127] Lukashev A.N., Shumilina E.Y., Belalov I.S., Ivanova O.E., Eremeeva T.P., Reznik V.I., Trotsenko O.E., Drexler J.F., Drosten C. (2014). Recombination strategies and evolutionary dynamics of the Human enterovirus A global gene pool. J. Gen. Virol..

[128] Egger D., Bienz K. (2002). Recombination of Poliovirus RNA Proceeds in Mixed Replication Complexes Originating from Distinct Replication Start Sites. J. Virol..

[129] Kirkegaard K., Baltimore D. (1986). The mechanism of RNA recombination in poliovirus. Cell.

[130] Chetverin A.B. (1999). The puzzle of RNA recombination. FEBS Lett..

[131] Gmyl A.P., Belousov E.V., Maslova S.V., Khitrina E.V., Chetverin A.B., Agol V.I. (1999). Nonreplicative RNA Recombination in Poliovirus. J. Virol..

[132] Kyriakopoulou Z., Pliaka V., Amoutzias G.D., Markoulatos P. (2015). Recombination among human non-polio enteroviruses: Implications for epidemiology and evolution. Virus Genes.

[133] Muslin C., Joffret M.-L., Pelletier I., Blondel B., Delpeyroux F. (2015). Evolution and Emergence of Enteroviruses through Intra- and Inter-species Recombination: Plasticity and Phenotypic Impact of Modular Genetic Exchanges in the 5′ Untranslated Region. PLoS Pathog..

[134] Yozwiak N.L., Skewes-Cox P., Gordon A., Saborio S., Kuan G., Balmaseda A., Ganem D., Harris E., DeRisi J.L. (2010). Human Enterovirus 109: A Novel Interspecies Recombinant Enterovirus Isolated from a Case of Acute Pediatric Respiratory Illness in Nicaragua. J. Virol..

[135] Santti J., Hyypia T., Kinnunen L., Salminen M. (1999). Evidence of Recombination among Enteroviruses. J. Virol..

[136] Martin D.P., Murrell B., Golden M., Khoosal A., Muhire B. (2015). RDP4: Detection and analysis of recombination patterns in virus genomes. Virus Evol..

[137] Fu L., Niu B., Zhu Z., Wu S., Li W. (2012). CD-HIT: Accelerated for clustering the next-generation sequencing data. Bioinformatics.

[138] Yoke-Fun C., Abubakar S. (2006). Phylogenetic evidence for inter-typic recombination in the emergence of human enterovirus 71 subgenotypes. BMC Microbiol..

[139] Majumdar M., Sharif S., Klapsa D., Wilton T., Alam M.M., Fernandez-Garcia M.D., Rehman L., Mujtaba G., McAllister G., Harvala H. (2018). Environmental Surveillance Reveals Complex Enterovirus Circulation Patterns in Human Populations. Open Forum Infect. Dis..

[140] Pickett B.E., Sadat E.L., Zhang Y., Noronha J.M., Squires R.B., Hunt V., Liu M., Kumar S., Zaremba S., Gu Z. (2012). ViPR: An open bioinformatics database and analysis resource for virology research. Nucleic Acids Res..

[141] Bo Y.-C., Song C., Wang J.-F., Li X.-W. (2014). Using an autologistic regression model to identify spatial risk factors and spatial risk patterns of hand, foot and mouth disease (HFMD) in Mainland China. BMC Public Health.

[142] Khetsuriani N., Lamonte-Fowlkes A., Oberst S., Pallansch M.A. (2006). Enterovirus surveillance—United States, 1970–2005. MMWR Surveil. Summ..

[143] Tee K.K., Lam T.T., Chan Y.F., Bible J.M., Kamarulzaman A., Tong C.Y., Takebe Y., Pybus O.G. (2010). Evolutionary Genetics of Human Enterovirus 71: Origin, Population Dynamics, Natural Selection, and Seasonal Periodicity of the VP1 Gene. J. Virol..

[144] Tokarz R., Firth C., Madhi S.A., Howie S.R., Wu W., Sall A.A., Haq S., Briese T., Lipkin W.I. (2012). Worldwide emergence of multiple clades of enterovirus 68. J. Gen. Virol..

[145] Yarmolskaya M.S., Shumilina E.Y., Ivanova O.E., Drexler J.F., Lukashev A. (2015). Molecular epidemiology of echoviruses 11 and 30 in Russia: Different properties of genotypes within an enterovirus serotype. Infect. Genet. Evol..

[146] Oberste M.S., Maher K., Kennett M.L., Campbell J.J., Carpenter M.S., Schnurr D., Pallansch M.A. (1999). Molecular Epidemiology and Genetic Diversity of Echovirus Type 30 (E30): Genotypes Correlate with Temporal Dynamics of E30 Isolation. J. Clin. Microbiol..

[147] Savolainen C., Hovi T., Mulders M.N. (2001). Molecular epidemiology of echovirus 30 in Europe: Succession of dominant sublineages within a single major genotype. Arch. Virol..

[148] Brown B.A., Nix W.A., Sheth M., Frace M., Oberste M.S. (2014). Seven Strains of Enterovirus D68 Detected in the United States during the 2014 Severe Respiratory Disease Outbreak. Genome Announc..

[149] Poelman R., Schuffenecker I., Van Leer-Buter C., Josset L., Niesters H.G., Lina B. (2015). European surveillance for enterovirus D68 during the emerging North-American outbreak in 2014. J. Clin. Virol..

[150] Midgley C.M., Watson J.T., Nix W.A., Curns A.T., Rogers S.L., Brown B.A., Conover C., Dominguez S.R., Feikin D.R., Gray S. (2015). Severe respiratory illness associated with a nationwide outbreak of enterovirus D68 in the USA (2014): A descriptive epidemiological investigation. Lancet Respir. Med..

[151] Oh M.-D., Park S., Choi Y., Kim H., Lee K., Park W., Yoo Y., Kim E.-C., Choe K. (2003). Acute Hemorrhagic Conjunctivitis Caused by Coxsackievirus A24 Variant, South Korea, 2002. Emerg. Infect. Dis..

[152] Pons-Salort M., Oberste M.S., Pallansch M.A., Abedi G.R., Takahashi S., Grenfell B.T., Grassly N.C. (2018). The seasonality of nonpolio enteroviruses in the United States: Patterns and drivers. Proc. Natl. Acad. Sci. USA.

[153] Yang B., Liu F., Liao Q., Wu P., Chang Z., Huang J., Long L., Luo L., Li Y., Leung G.M. (2017). Epidemiology of hand, foot and mouth disease in China, 2008 to 2015 prior to the introduction of EV-A71 vaccine. Eurosurveillance.

[154] Podin Y., Gias E.L., Ong F., Leong Y.W., Yee S.F., Yusof M.A., Perera D., Teo B., Wee T.-Y., Yao S.-K. (2006). Sentinel surveillance for human enterovirus 71 in Sarawak, Malaysia: Lessons from the first 7 years. BMC Public Health.

[155] National Institute of Infectious Diseases (2012). Hand, Foot and Mouth Disease in Japan, 2002–2011. https://www.niid.go.jp/niid/en/basic-science/865-iasr/1735-tpc385.html.

[156] Mladenova Z., Buttinelli G., Dikova A., Stoyanova A., Troyancheva M., Komitova R., Stoycheva M., Pekova L., Parmakova K., Fiore L. (2014). Aseptic meningitis outbreak caused by echovirus 30 in two regions in Bulgaria, May–August 2012. Epidemiol. Infect..

[157] Kim H., Kang B., Hwang S., Hong J. (2012). Epidemics of viral meningitis caused by echovirus 6 and 30 in Korea in 2008. Virol. J..

[158] Rudolph H., Dernbach R.P., Walka M., Rey-Hinterkopf P., Melichar V., Muschiol E., Schweitzer-Krantz S., Richter J.W., Weiss C., Böttcher S. (2017). Comparison of clinical and laboratory characteristics during two major paediatric meningitis outbreaks of echovirus 30 and other non-polio enteroviruses in Germany in 2008 and 2013. Eur. J. Clin. Microbiol. Infect. Dis..

[159] Brinkman N.E., Fout G.S., Keely S.P. (2017). Retrospective Surveillance of Wastewater To Examine Seasonal Dynamics of Enterovirus Infections. mSphere.

[160] Faleye T.O.C., Adewumi M.O., Japhet M.O., David O.M., Oluyege A.O., Adeniji J.A., Famurewa O. (2017). Non-polio enteroviruses in faeces of children diagnosed with acute flaccid paralysis in Nigeria. Virol. J..

[161] Zhang Y., Hong M., Sun Q., Zhu S., Tsewang, Li X., Yan D., Wang D., Xu W. (2014). Molecular typing and characterization of a new serotype of human enterovirus (EV-B111) identified in China. Virus Res..

[162] Adeniji J.A., Ayeni F.A., Ibrahim A., Tijani K.A., Faleye T.O.C., Adewumi M.O. (2017). Comparison of Algorithms for the Detection of Enteroviruses in Stool Specimens from Children Diagnosed with Acute Flaccid Paralysis. J. Pathog..

[163] Horner L.M., Poulter M.D., Brenton J.N., Turner R.B. (2015). Acute Flaccid Paralysis Associated with Novel Enterovirus C105. Emerg. Infect. Dis..

[164] Maan H.S., Dhole T.N., Chowdhary R. (2019). Identification and characterization of nonpolio enterovirus associated with nonpolio-acute flaccid paralysis in polio endemic state of Uttar Pradesh, Northern India. PLoS ONE.

[165] Barnadas C., Midgley S.E., Skov M.N., Jensen L., Poulsen M.W., Fischer T.K. (2017). An enhanced Enterovirus surveillance system allows identification and characterization of rare and emerging respiratory enteroviruses in Denmark, 2015–2016. J. Clin. Virol..

[166] Donbraye E., Olasunkanmi O.I., Opabode B.A., Ishola T.R., Faleye T.O.C., Adewumi O.M., Adeniji J.A. (2018). Abundance of enterovirus C in RD-L20B cell culture-negative stool samples from acute flaccid paralysis cases in Nigeria is geographically defined. J. Med. Microbiol..

