# Supplementary material for: Epidemiology and Sequence-Based Evolutionary Analysis of Circulating Non-Polio Enteroviruses
Source: Microorganisms. 2020 Nov 25;8(12):1856. doi: 10.3390/microorganisms8121856 (PMC7759938; doi:10.3390/microorganisms8121856)
Supplement: Supplementary file 1 [file microorganisms-08-01856-s001.pdf]

Table S1. Number of genomic sequence records for all non-polio EVs.

Number of sequences*			
All time	2014 to 2019	Species	Type
7174	2890	A	Coxsackievirus A6
12720	2413	A	Enterovirus A71
5318	1627	A	Coxsackievirus A16
2068	742	A	Coxsackievirus A10
974	435	A	Coxsackievirus A4
418	139	A	Coxsackievirus A2
275	111	A	Coxsackievirus A5
175	28	A	Coxsackievirus A8
54	11	A	Coxsackievirus A12
20	8	A	Enterovirus A119
72	6	A	Coxsackievirus A14
11	5	A	Enterovirus A120
31	1	A	Coxsackievirus A7
13	0	A	Coxsackievirus A3
54	0	A	Enterovirus A76
16	0	A	Enterovirus A89
51	0	A	Enterovirus A90
4	0	A	Enterovirus A91
41	0	A	Enterovirus A92
1	0	A	Enterovirus A114
0	0	A	Enterovirus A121
2211	653	B	Echovirus 11
1850	612	B	Coxsackievirus B5
4402	516	B	Echovirus 30
2139	497	B	Echovirus 6
569	211	B	Echovirus 18
1244	194	B	Coxsackievirus B3
551	178	B	Echovirus 25
238	166	B	Echovirus 16
1100	129	B	Coxsackievirus B4
803	106	B	Echovirus 7
778	97	B	Echovirus 9
430	90	B	Echovirus 3
549	75	B	Coxsackievirus B2
539	69	B	Coxsackievirus B1
620	68	B	Coxsackievirus A9
365	54	B	Echovirus 19
957	45	B	Echovirus 13
188	34	B	Echovirus 20
151	33	B	Echovirus 1
137	31	B	Echovirus 21
258	27	B	Echovirus 14
142	21	B	Echovirus 24
150	20	B	Echovirus 29

122	18	B	Echovirus 5
194	18	B	Echovirus 33
160	10	B	Echovirus 12
33	8	B	Enterovirus B73
87	7	B	Echovirus 2
30	7	B	Echovirus 31
126	5	B	Enterovirus B75
14	5	B	Enterovirus B88
44	4	B	Echovirus 15
130	3	B	Echovirus 4
9	3	B	Enterovirus B78
105	2	B	Coxsackievirus B6
57	2	B	Echovirus 17
44	2	B	Echovirus 27
28	2	B	Echovirus 32
44	2	B	Enterovirus B69
59	1	B	Enterovirus B80
12	1	B	Enterovirus B84
2	1	B	Enterovirus B111
24	0	B	Echovirus 26
25	0	B	Enterovirus B74
16	0	B	Enterovirus B77
2	0	B	Enterovirus B79
9	0	B	Enterovirus B81
6	0	B	Enterovirus B82
38	0	B	Enterovirus B83
36	0	B	Enterovirus B85
5	0	B	Enterovirus B86
4	0	B	Enterovirus B87
8	0	B	Enterovirus B93
0	0	B	Enterovirus B97
0	0	B	Enterovirus B98
0	0	B	Enterovirus B100
0	0	B	Enterovirus B101
0	0	B	Enterovirus B106
0	0	B	Enterovirus B107
974	157	C	Coxsackievirus A24
258	33	C	Coxsackievirus A13
154	27	C	Coxsackievirus A21
133	27	C	Enterovirus C99
101	26	C	Enterovirus C96
153	24	C	Coxsackievirus A20
56	21	C	Coxsackievirus A22
25	10	C	Enterovirus C109
23	8	C	Enterovirus C116
14	6	C	Enterovirus C105
40	5	C	Coxsackievirus A1

53	5	C	Coxsackievirus A11
27	5	C	Coxsackievirus A19
73	3	C	Coxsackievirus A17
27	2	C	Enterovirus C104
5	1	C	Enterovirus C95
4	1	C	Enterovirus C102
7	1	C	Enterovirus C117
5	0	C	Enterovirus C118
3841	2895	D	Enterovirus D68
45	0	D	Enterovirus D70
8	1	D	Enterovirus D94
8	1	D	Enterovirus D111

* The number of sequence records for each enterovirus type in ViPR (Table 1) was determined. The number of sequences was determined by collecting references to each NPEV sequence in the ViPR database. Search was performed using the "Gene/Protein Search," viruses Enterovirus A-D were included in the search. Search type included the "Gene Product Name" term "VP1". Data collection performed October 9th, 2020. Each reference was counted by "virus type" name and all aliases added together using Microsoft Excel
